# Effects of Age, Sex, and Exercise on Measurement of Serum CTnI Levels and Some Parameters Related to the Cardiovascular Capacity of Caspian Horses

**DOI:** 10.1002/vms3.70202

**Published:** 2025-03-10

**Authors:** Hossein Mehrazin, Mehdi Sakha, Shahabeddin Safi

**Affiliations:** ^1^ Department of Clinical Science School of Veterinary Medicine Science and research branch,Islamic Azad University Tehran Iran; ^2^ Department of Pathobiology Faculty of Veterinary Medicine Science and Research Branch Islamic Azad University Tehran Iran

**Keywords:** cardiac biomarkers, cardiac troponin, horse, packed cell volume

## Abstract

Due to their high specificity and exclusive cardiac myocyte sensitivity, cardiac troponins T and I (cTnT, cTnI) are currently regarded as ideal biomarkers to identify cardiomyocyte damage, myocardial injury, myocardial infarction, and chronic heart failure. In fact, cTnI is considered the most reliable biomarker for diagnosing heart‐related issues. This study aimed to investigate the effects of age, gender, and exercise training on serum cTnI levels and various parameters related to the cardiovascular capacity of Caspian horses. For this purpose, 50 adult Caspian horses over 3 years old, both male and female, were selected from horse breeding centres and clubs in the provinces of Tehran, Alborz, and Gilan. To account for age‐related differences, the horses were divided into three groups: Group A (less than 5 years), Group B (between 5 and 10 years), and Group C (over 10 years). To measure cTnI levels, 10 mL of blood was collected from the jugular vein of each horse using a venoject blood collection tube before exercise and another 10 mL 2 h post‐exercise. The samples were refrigerated and centrifuged for 30 min after collection. Two millilitres of serum obtained from each horse at both time points was stored at −20°C for subsequent analysis. Troponin I levels were measured in the laboratory using the electrochemical luminescence (ECL) method. The results of this study reveal for the first time that the normal average of serum cTnI, packed cell volume (PCV), and blood haemoglobin (Hb) levels in Caspian horses is 2.5 ng/L, 35.52%, and 12.1%, respectively. Furthermore, the findings indicate that exercise significantly increases serum levels of cTnI, PCV, and blood haemoglobin; however, age and gender did not appear to affect these measured parameters. Therefore, it can be concluded that exercise stimulates the release of troponin due to myocardial injury.

## Graphical abstract

The results of this study reveal for the first time that the normal average of serum cTnI, packed cell volume (PCV), and blood haemoglobin (Hb) levels in Caspian horses are 2.5 ng/L, 35.52%, and 12.1%, respectively. Furthermore, the findings indicate that exercise significantly increases serum levels of cTnI, PCV, and blood haemoglobin; however, age and gender did not appear to affect these measured parameters.

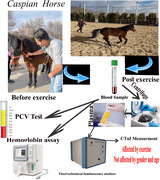

## 1 Introduction

The relationship between cardiovascular responses and exercise in equine sports medicine has been the subject of recent exploration (Fazio et al. 2023). The energy required for the work and exercise of a horse's muscles is generated through metabolic processes occurring in the muscles, liver, and adipose tissue (Votion et al. 2007). The cardiovascular system plays a crucial role in regulating physical activity (Ferlazzo et al. 2020). Cardiac activity regulation is a natural process that relies on the rhythmic actions of the autonomic nervous system (ANS), particularly the sympathetic and parasympathetic branches. Additionally, the vagus nerve, the respiratory cycle, and fluctuations in neuro‐hormonal components released into the bloodstream also influence the cardiovascular system (Janczarek et al. 2020). The vagus nerve affects the sinoatrial node via the neurotransmitter acetylcholine, which alters iron and calcium flow in cardiac cells, ultimately slowing conduction (Nunan, Sandercock, and Brodie 2010; Howland 2014).

One important indicator of heart health is the packed cell volume (PCV) test, which assesses vascular capacity and the ability to mobilize blood cells from the spleen, as well as overall cardiovascular capacity (Mihisy et al. 2017). In addition, according to the study of Mozos et al. (2015), a decrease of 1 g/dL in the level of Hb is an independent risk factor for cardiac complications and mortality, and different forms of anaemia are associated with cardiovascular complications.

In horses, cardiovascular problems rank as the third leading cause of poor performance, following musculoskeletal and respiratory disorders (Alberti et al. 2021). Heart disease is recognized as a potential cause of sudden death or diminished performance in these animals (de Solis et al. 2018). Diagnosing heart disease in horses is challenging due to the need for specific cardiac signals (Patteson 2010). Clinically, equine cardiac diseases may present with non‐specific symptoms such as myalgia, tachycardia, fever, and reluctance to move (Ayvazoğlu et al. 2023). The application of cardiac biomarkers in the clinical evaluation of critically ill patients is currently being investigated and shows promise, with their use in veterinary medicine increasing rapidly (Jaffe, Babuin, and Apple 2006). Notably, elevated levels of cardiac biomarkers can also be associated with high‐intensity exercise (Flethøj et al. 2016). Several specific cardiac biomarkers, including cardiac troponins T and I (cTnT and cTnI), natriuretic peptides, creatine kinase (CK), and myoglobin (MB), have been employed to assess cardiac function in horses within clinical contexts (Ayvazoğlu et al. 2023).

Cardiac troponin (cTn) is a crucial component of the thin actin filaments within the sarcomere of striated muscle, playing a vital role in the contractile and excitatory activities of the heart (Guma 2019). The cardiac troponin isoform comprises three subunits: T (tropomyosin binding), I (inhibitory), and C (calcium binding) (Johnston et al. 2019). When cardiac muscle cells are damaged, cTnI and cTnT leak into the cytosol, leading to their release into the bloodstream (Katrukha and Katrukha 2021). Due to their high sensitivity and absolute specificity for cardiomyocytes, cTnT and cTnI are currently regarded as ideal biomarkers for identifying cardiomyocyte damage, myocardial injury (including myocardial necrosis), myocardial infarction, and chronic heart failure. Furthermore, all clinical guidelines recommend measuring cTn as the reference standard for differentiating acute coronary syndromes (Clerico et al. 2021; Parsanathan Jain 2020). Although troponin isoforms are also found in skeletal muscle, in equine medicine, cTnI is recognized as the most effective biomarker for diagnosing cardiac issues (van de Locht et al. 2021; Rishniw Simpson 2005). Human and equine cTnI are highly similar and utilize the same measurement techniques. The standard normal value for cTnI in horses is 0.03 ng/mL (Nath et al. 2012). Levels above this threshold indicate varying degrees of cardiac muscle dysfunction (Kraus et al. 2013).

Physiological adaptations of the heart can lead to an increase in heart chamber size. Factors such as age, sex, exercise, body weight, and, in certain species, breed significantly influence heart dimensions (Trachsel et al. 2016). While numerous studies have validated the impact of various factors on cardiac function in racehorses, research on other horse breeds has been comparatively limited (Younes et al. 2016; Adamu et al. 2013; et al., 2006).

The Caspian horse, or Miniature Caspian horse, is recognized as one of the oldest horse breeds in the world and is considered the first ancestor of warm‐blooded horses, dating back ∼5000 years. This horse is native to the Caspian Sea region, which was only in Iran until 1345 (Willekes 2013). The Caspian horse is regarded as a valuable genetic resource and an important economic species, making the study of its natural physiology and health particularly significant. Consequently, this study aims to investigate the effects of age, sex, and exercise on serum cTnI levels and various parameters related to cardiovascular capacity in the Caspian horse.

## 2 Materials and Methods

### 2.1 Animals of Study

The statistical population size was determined using the standard method (Pfeiffer 2010). Fifty adult Caspian horses (25 males and 25 females) with an average age of more than 3 years were selected from breeding centres and horse clubs in the provinces of Tehran, Alborz, and Gilan and evaluated clinically (by auscultation of breathing and heartbeat with a stethoscope and electrocardiogram). Before starting the study, their health was determined by complete blood count (CBC), blood biochemistry, kidney, and liver factors (alanine transferase and aspartate transaminase) enzymes, etc.). Unhealthy horses were excluded from the study. The studied horses were kept in horse breeding centres under regular and normal training, and none of them was subjected to intensive training or sports activity. To account for the interference of age in the study, the Caspian horses were divided into three groups: less than 5 years in Group A, from 5 to 10 years in Group B, and more than 10 years in Group C. We tried to have equal numbers of both sexes in each group as much as possible (Table 1).

**TABLE 1:** Characteristics table of studied animals.

Group	Number of horses (male‐ female)	Animals mean age (mean ± SD)	Castrate or non‐castrate	Horses' weight (mean ± SD)	Presence or history of respiratory or cardiovascular disease
Group A	17 (9–8)	4.10 ± 0.75	Non‐castrate	206.82 ± 3.46	No sign/no history
Group B	17 (8–9)	8.19 ± 0.96	Non‐castrate	216.47 ± 5.93	No sign/no history
Group C	16 (8–8)	12.2 ± 3.47	Non‐castrate	225.25 ± 5.59	No sign/no history
Total	50 (25–25)	8.11 ± 3.47	All non‐castrate	216 ± 9.06	All animals were healthy

**TABLE 2:** Statistical analysis related to the various tests studied.

No.	Analysis	Pre‐post exercise	Age intervention	Gender intervention
1	CTnI	Paired *T*‐test and confidence interval (CI)	One‐way ANOVA (Welch's test)	Two‐sample *T*‐test and CI
2	PCV	Two‐way paired *T*‐test and CI	One‐way ANOVA (Welch's test)	Two‐sample *T*‐test and CI
3	Hb	Two‐sample *T*‐test and CI	One‐way ANOVA (Welch's test)	Two‐sample *T*‐test and CI

### 2.2 Sampling

Sampling of each animal was performed by a veterinarian under aseptic conditions and in a standard manner. For this, after the physical immobilization of the animal, the neck area was disinfected with cotton and alcohol (70% ethanol); then, the blood sample was taken in two stages before (before starting to longeing the animal) and after the exercise performed in a similar way. First, we longed the horse, and the training consisted of walking for 5 min to prepare and then galloping at maximum speed (about 6 m per second) and covering a distance of 800 m.

### 2.3 PCV and Haemoglobin Measurement Test

To conduct the PCV and haemoglobin measurement test, 2 mL of blood was collected from each horse using a 22‐gauge needle and an EDTA K2 blood collection tube (Purple Top) both before exercise and immediately after exercise. The collected blood samples were then sent to the biochemistry laboratory at the Faculty of Veterinary Medicine. In the laboratory, the PCV of the samples was measured using a micro‐haematocrit instrument (Hawksley Co., UK). For haemoglobin measurement, a Coulter counter (MEK‐6450; Nihon Koden, Japan) was employed.

### 2.4 Measurement of Serum Troponin I (CTnI) Level

To measure the amount of CTnI, 10 mL of blood was collected from the jugular vein of each horse with a venoject collection tube (Top red) before the exercise and ​​10 mL 2 h after the exercise. The samples were refrigerated and centrifuged for 30 min after collection. A total of 2 mL of serum obtained from each animal at each stage was stored at −20°C for analysis. Troponin I was measured in the laboratory with serum separated by the electrochemical‐luminescence (ECL) method (Regan, O'Kennedy, and Collins 2018).

### 2.5 Data Analysis

Data analysis was performed using one‐way analysis of variance (ANOVA) to evaluate the differences in troponin I, PCV, and haemoglobin levels before and after exercise in Caspian horses. The normality of the distribution of parameters was assessed using the Kolmogorov–Smirnov test. Post hoc comparisons were made to identify significant differences. The analytical methods are detailed in Table 2. Results are presented as mean ± standard deviation, with a *p* value of < 0.05 considered statistically significant. All calculations were conducted using SPSS version 24.

## 3 Results

### 3.1 Measurement of Serum CTnI

**FIGURE 1:**
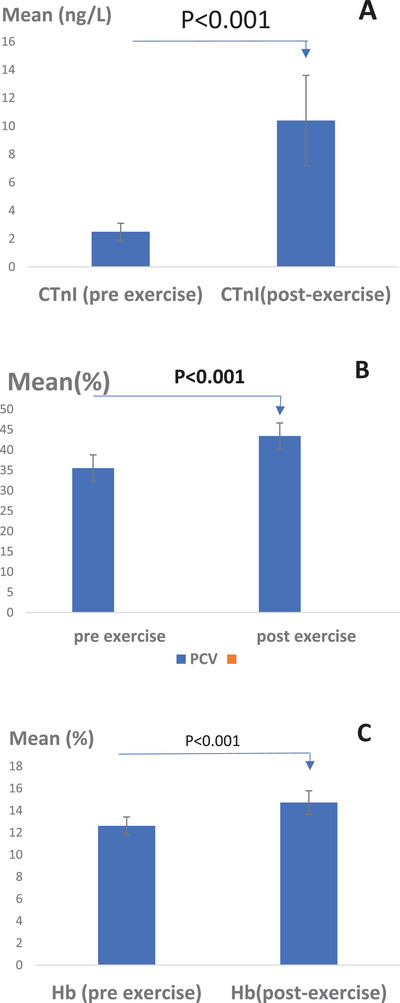
(A) The average amount of serum cTnI in Caspian horses before (CTnI0) and after exercise (CTnI1). Statistically significant difference was observed (*p* < 0.05). (B) Average percentage of PCV in Caspian horses pre‐ and post‐exercise. Statistically significant difference was observed (*p* value < 0.001). (C) Average percentage of Hb in Caspian horses pre‐ and post‐exercise. Statistically significant difference was observed (*p* value < 0.001). Scale: percentage.

The amount of CTnI was measured in the normal state (before the exercise) and taking into account the interferences related to age and sex before and after the exercise (Figure 1A). The average cTnI level in the horses under normal conditions was found to be 2.5 ± 0.6 ng/L. After exercise, the average cTnI concentration increased significantly to 10.4 ± 3.2 ng/L. Statistical analysis revealed a significant difference in serum cTnI levels before and after exercise (*p* < 0.05, *p* = 0.001).

**FIGURE 2:**
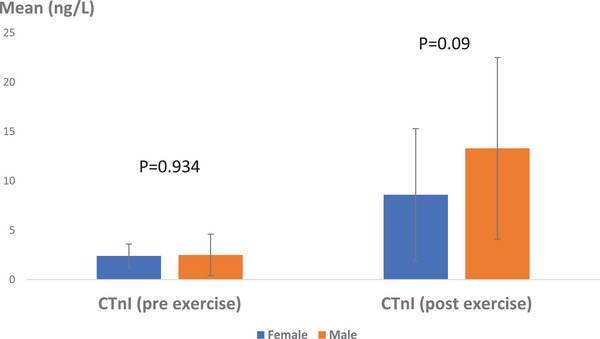
Mean serum CTnI levels in Caspian horses, taking into account the intervention of sex pre‐ and post‐exercise. No statistically significant difference was observed (*p* > 0.05). Scale: ng/L.

When examining the influence of gender on cTnI levels, the pre‐exercise average was calculated to be 2.4 ± 1.2 ng/L in females and 2.5 ± 2.1 ng/L in males (Figure 2). The statistical analysis indicated that gender did not significantly affect the mean cTnI level prior to exercise (*p* > 0.05, *p* = 0.943). Post‐exercise measurements showed an average cTnI level of 8.6 ± 6.7 ng/L in females and 13.3 ± 9.2 ng/L in males; however, statistical analysis revealed no significant difference between sexes after exercise (*p* > 0.05, *p* = 0.090).

**FIGURE 3:**
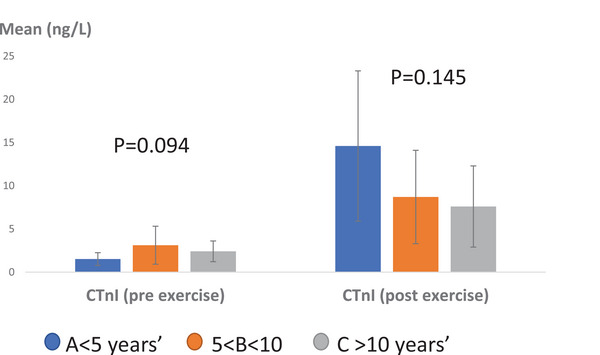
Mean serum CTnI levels in Caspian horses pre‐ and post‐exercise considering the intervention of age. No statistically significant difference was observed (*p* > 0.05). Scale: ng/L.

Regarding the impact of age, the average pre‐exercise serum cTnI levels were recorded as follows: Group A had 1.52 ± 0.74 ng/L, Group B had 3.12 ± 2.2 ng/L, and Group C had 2.41 ± 1.2 ng/L (Figure 3). Statistical analysis did not indicate any significant differences among these groups (*p* > 0.05, *p* = 0.094). After exercise, the average serum cTnI levels were measured at 14.6 ± 8.7 ng/L in Group A, 8.7 ± 5.4 ng/L in Group B, and 7.6 ± 4.7 ng/L in Group C, with no statistically significant differences observed (*p* > 0.05, *p* = 0.145).

### 3.2 Measurement of Haematocrit (PCV)

The results of the average percentage of blood haematocrit of Caspian horses were measured and recorded before and after exercise under normal conditions and taking into account the interferences related to age and sex (Figure 1B). The average PCV before exercise was found to be 35.52 ± 3.25%, which increased to 43.42 ± 3.20% after exercise. Statistical analysis indicated a significant difference in PCV levels before and after exercise (*p* < 0.05, *p* = 0.001).

**FIGURE 4:**
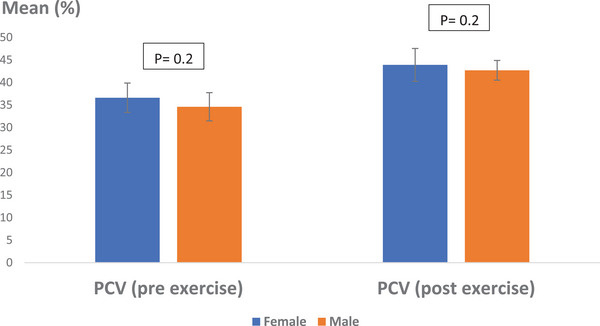
Average percentage of PCV in Caspian horses pre‐ and post‐exercise, considering the intervention of gender. No statistically significant difference was observed (*p* > 0.05). Scale: percentage.

When examining the influence of gender on PCV results, the pre‐exercise average was 36.06 ± 3.27% for female Caspian horses and 34.60 ± 3.13% for males (Figure 4). The pre‐exercise data did not reveal a statistically significant difference based on gender (*p* > 0.05, *p* = 0.202). After exercise, the average PCV in females was measured at 43.89 ± 3.65%, while in males it was 42.62 ± 2.19%. The statistical analysis of post‐exercise data also showed no significant difference related to gender (*p* > 0.05, *p* = 0.208).

**FIGURE 5:**
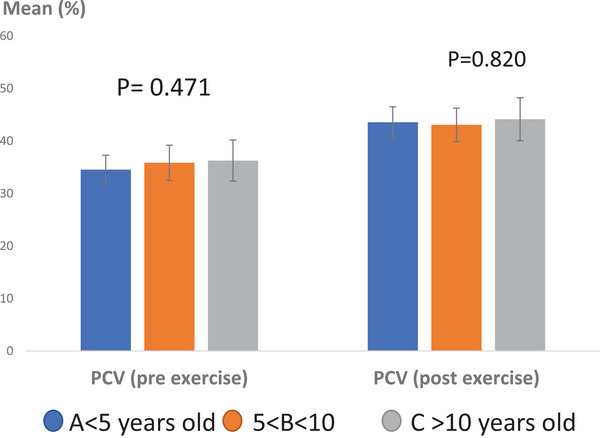
Average percentage of PCV in Caspian horses pre‐ and post‐exercise, considering the intervention of age. No statistically significant difference was observed (*p* > 0.05). Scale: percentage.

Regarding the effect of age on PCV levels, the average pre‐exercise PCV was recorded as 34.55 ± 2.74% in Group A, 35.86 ± 3.35% in Group B, and 36.28 ± 3.92% in Group C (Figure 5). No statistically significant differences were observed among these groups before exercise (*p* > 0.05, *p* = 0.471). After exercise, the average PCV for the groups was recorded as follows: 43.58 ± 2.94% in Group A, 43.07 ± 3.19% in Group B, and 44.15 ± 4.09% in Group C. Data analysis indicated no significant differences post‐exercise concerning age groups (*p* > 0.05, *p* = 0.820).

### 3.3 Measurement of Haemoglobin

The average haemoglobin (Hb) percentage in the blood of Caspian horses was measured both before and after exercise, taking into account the potential influences of age and gender. The average Hb percentage prior to exercise was found to be 12.61 ± 0.8%, which increased to 14.72 ± 1.06% following exercise. Statistical analysis indicated a significant difference in Hb levels before and after exercise (*p* < 0.05, *p* < 0.001) (Figure 1C).

**FIGURE 6:**
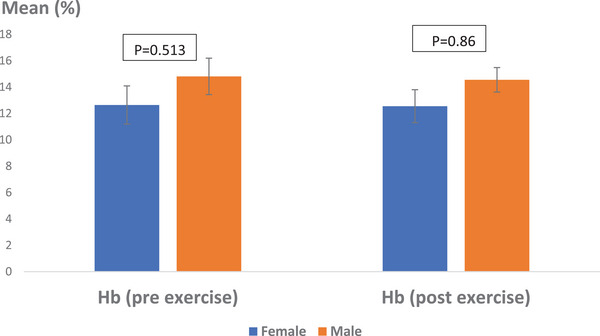
Average percentage of Hb in Caspian horses pre‐ and post‐exercise, taking into account sex intervention. No statistically significant difference was observed (*p* > 0.05). Scale: percentage.

In terms of gender differences, the average Hb percentage in female Caspian horses before exercise was 12.64 ± 1.45%, while in males, it was 12.55 ± 1.25%. The pre‐exercise data did not reveal a statistically significant difference based on gender (*p* > 0.05, *p* = 0.513). After exercise, the average Hb percentage for females was 14.81 ± 1.38%, compared to 14.55 ± 0.93% for males. Statistical analysis of the post‐exercise data also showed no significant difference related to gender (*p* > 0.05, *p* = 0.860) (Figure 6).

**FIGURE 7:**
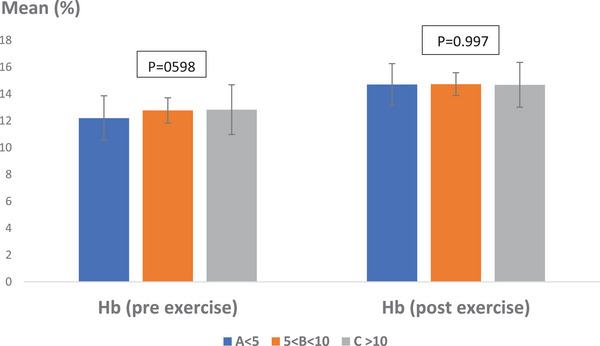
Average percentage of Hb in Caspian horses pre‐ and post‐exercise, taking into account age intervention. No statistically significant difference was observed (*p* > 0.05). Scale: percentage.

Regarding the effect of age on Hb levels, the average Hb percentage before exercise was recorded as 12.2 ± 1.66% in Group A, 12.77 ± 0.94% in Group B, and 12.83 ± 1.85% in Group C. After exercise, the average Hb percentages were 14.7 ± 1.56% for Group A, 14.73 ± 0.85% for Group B, and 14.68 ± 1.67% for Group C (*p* > 0.05, *p* = 0.598). No statistically significant differences were observed in Hb levels before and after exercise when analyzed by age group (*p* > 0.05, *p* = 0.997) (Figure 7).

## 4 Discussion

Evaluating and determining cardiovascular capacity in horses are crucial due to its direct correlation with health and performance. The measurement of CTnI has been conducted in various horse breeds and established reference standards for heart health (Reef et al. 2014; Shih 2019; Poole et al. 2020; Nostell and Häggström 2008; Serra et al. 2010; Phillips et al. 2003). These studies successfully identified heart disease using CTnI and facilitated the selection of more efficient horses for breeding purposes (Van Der Vekens et al. 2015). Despite the significance of this research, no studies have been conducted globally to assess cardiac capacity, establish reference standards for CTnI, or determine normal interval levels and their variations after exercise specifically in Caspian horses. This study aims to fill that gap. Notably, many countries have recognized the importance of the Caspian horse as a genetic and athletic resource, particularly for children, leading to an increasing interest in utilizing the abilities of this breed (Willekes 2013). As the primary origin of this breed, Iran should also be acknowledged as a scientific reference point (Sadeghi et al. 2019; Zandi, Javaremi, and Pakde 2014).

This research involved measuring CTnI concentrations in 50 Caspian horses prior to exercise. Initially, we established the normal average for CTnI levels. We then assessed cardiac health by monitoring changes in CTnI and evaluating PCV before and after exercise to gauge cardiac system capacity. Importantly, age and gender variables were taken into account throughout the study. Cardiac troponins are highly specific biomarkers indicative of myocardial cell damage and are widely utilized in both human and veterinary medicine to identify cardiac pathologies in horses. They play a vital role in diagnosing physiological changes and diseases (Shields et al. 2018). Among these biomarkers, CTnI and cTnT are recognized for their specificity; however, due to its lower molecular weight, CTnI is considered more sensitive and specific (Kırbaş et al. 2021; Carretón, Morchón, and Montoya‐Alonso 2017).

The most significant finding of this research was the establishment of the normal average for CTnI in the blood of Caspian horses, which was measured at 2.5 ng/L. Determining this normal average is essential for documenting and analyzing changes in cardiac health and identifying pathological conditions, particularly within specific breeds. Furthermore, as CTnI is acknowledged as a key biomarker for cardiac diseases, establishing its normal average in Caspian horses is of particular importance (Du et al. 2021; Jaffe, Babuin, and Apple 2006; Nadir et al. 2012; Spratt et al. 2005).

In studies examining serum cTnI levels in healthy horses, a range of 0.01–0.03 ng/mL has been reported (Spratt et al. 2005; Ayvazoğlu et al. 2023; Phillips et al. 2003; Nath et al. 2012). The different levels observed in Caspian horses may be attributed to genetic, physiological, and nutritional factors (Luu et al. 2016; Van Der Vekens et al. 2012). As the research by Trachsel et al. (2016) indicates that various factors such as body size, weight, age, gender, and breed can influence equine cardiovascular health. In fact, this study highlights the effect of breed variation on serum cTnI levels in Caspian horses compared to other breeds.

In our study, serum cTnI levels demonstrated a significant increase after exercise, measuring at 10.4 ± 7.4 ng/L. It has been established that exercise can lead to transient myocardial damage or stress (Pourmohammad, Lida Moghaddam‐Banaem, and Karamnia Far 2020). Following cardiac damage, cTnI levels in the blood typically peak within a few hours. However, there is conflicting evidence regarding the immediate post‐exercise increase in cTnI levels (Chow et al. 2017; Hellings et al. 2020; Trachsel et al. 2013; Baker et al. 2019). Our findings support studies that indicate a significant rise in serum cTnI levels within the initial hours following exercise.

In human studies, significant differences exist in clinical characteristics between women and men with heart disease, which also affect the kinetics of cardiac biomarkers. Women generally exhibit lower levels of cardiac troponin compared to men. Various biological factors may explain these differences, including body composition, fat distribution, and hormonal influences (Cediel et al. 2021). Additionally, women are less susceptible to conditions such as arteriosclerosis, left ventricular hypertrophy, and cardiomyocyte apoptosis than men. However, it is important to consider the cardio protective effects of oestrogens, which can suppress cardiomyocyte apoptosis, as well as the potential role of testosterone in promoting cardiomyocyte hypertrophy and apoptosis (Papamitsou et al. 2011; Liu, Pedram, and Kim 2011; Iorga et al. 2017).

According to Ayvazoğlu et al. (2023), exercise induces the production of reactive oxygen species (ROS) in horses, and it is noteworthy that the ability of male and female horses to adapt to and repair damage differs. Veterinary literature has also reported that oestrogen secretion can have a protective effect on the heart (Kim et al. 1996). In a study by O'brien et al. (2006) involving 6‐ and 8‐month‐old male rats, serum cTnI levels were found to be 10 times higher than those in female rats of the same age. Furthermore, Pourmohammad, Lida Moghaddam‐Banaem, and Karamnia Far (2020) highlighted a significant effect of sex on cTnI concentrations in trained Arabian horses, noting that cTnI values in mares were significantly higher than those in stallions. In the present study, while considering the impact of sex on serum cTnI levels, no significant differences were observed before and after exercise. It is possible that significant differences may emerge if cTnI measurements are conducted at extended time intervals following exercise.

Age is recognized as a significant risk factor for cardiovascular disease and mortality (Cabiati et al. 2020). In humans, serum levels of cTnI tend to increase with age, particularly in individuals over 60 years old (Zethelius, Johnston, and Venge 2006). A study by O'brien et al. (2006) also found that increasing age was associated with elevated serum cTnI levels in rats. However, research by Van Der Vekens et al. (2015) indicated that age, weight, and height did not significantly influence troponin I and T levels in the serum of horses. Consistent with other studies on equines, our investigation found no significant differences in serum cTnI levels before and after exercise with respect to age.

In veterinary medicine, PCV is a widely used clinical test to assess the oxygen‐carrying capacity of blood (Marron, Hare, and Ramsey 2015). PCV measurement helps evaluate cardiovascular capacity and splenic contractility, predicting oxygen transport capability and providing valuable insights into fitness changes in horses. Typically, the haematocrit of a resting horse ranges from 36% to 42%. In our study, the haematocrit level of Caspian horse was measured at 35.52 ± 3.25% (Wood et al. 1992). This variation may be attributed to storage conditions, environmental factors, and genetic differences. Kang and Park (2017) reported a significant increase in PCV immediately after training in crossbreed horses; however, this value tended to decrease over time, especially in older horses. Similar findings have been reported in other studies that noted a significant increase in PCV following exercise (Đoković et al. 2021; Krumrych 2006; Macedo et al. 2017). McKeever et al. (2010) indicated that PCV levels increase with age; however, our study did not demonstrate a significant effect of age on blood PCV before or after exercise. Miknienė et al. (2013) observed important differences in blood haematocrit between male and female Žemaitukai horses, noting a decrease in red blood cells and haematocrit with advancing age.

In our study, exercise positively influenced blood Hb levels, but the effects of gender and age on average Hb percentage were not significant. The results indicated an increase in blood haemoglobin following exercise in horses. According to Kang and Park (2017), exercise capacity and efficiency can be evaluated through Hb concentration analysis, which serves as an indicator of oxygen transport capacity. The normal average blood Hb level in Caspian horses was measured at 12.61 g/dL. In contrast, Allaam et al. (2014) reported a normal blood Hb level of 15.45 g/dL in thoroughbred horses, noting a significant increase in Hb levels after exercise of 5 min. Racial differences among horse breeds can contribute to variations in these reported values (Takasu et al. 2013).

## 5 Conclusion

In general, the racial difference has a significant effect on the functioning of various organs and in the physiology of the body. In addition, maintenance conditions, food, and geographical and ecological factors are among the factors that affect health factors, cardiovascular capacity, and respiratory capacity, and so on. In this study, due to the importance of Caspian horse as an indigenous species of Iran, cardiovascular capacity and normal average serum troponin were evaluated. Considering the genetic importance of this precious species, it is hoped that more studies will be carried out.

## Author Contributions

Hossein Mehrazin: Data curation, project administration, supervision, visualization, writing. Mehdi Sakha: Conceptualization, data curation, formal analysis, methodology, writing. Shahabeddin Safi: Resources, methodology, validation, writing–review and editing.

## Ethics Statement

Ethical approval for this study was granted by the Local Ethics Committee of the Science and Research Branch of the Islamic Azad University (approval number: IR.IAU.SRB.REC.1402.015; 10 June 2023). All experiments and samples were performed in accordance with animal rights, and animals were not harmed during the clinical trial.

## Conflicts of Interest

The authors declare no conflicts of interest.

### Peer Review

The peer review history for this article is available at https://publons.com/publon/10.1002/vms3.70202.

## Data Availability

The data that support the findings of this study are available from the corresponding author upon reasonable request.
